# Study on the Hydration of α-Pinene Catalyzed by α-Hydroxycarboxylic Acid–Boric Acid Composite Catalysts

**DOI:** 10.3390/molecules28073202

**Published:** 2023-04-04

**Authors:** Zhonglei Meng, Rongxiu Qin, Rusi Wen, Guiqing Li, Zhongyun Liang, Junkang Xie, Zhangqi Yang, Yonghong Zhou

**Affiliations:** 1 Guangxi Key Laboratory of Superior Timber Trees Resource Cultivation, Guangxi Forestry Research Institute, Nanning 530002, China; 2 Institute of Chemical Industry of Forest Products, Chinese Academy of Forestry (CAF), Nanjing 210042, China

**Keywords:** α-hydroxycarboxylic acid–boric acid complexes, green catalyst, hydration of α-pinene, solvent-free, terpineol, *p*-menthane-1,8-diol monohydrate

## Abstract

In this study, seven types of α-hydroxycarboxylic acids were selected to form composite catalysts with boric acid, and their catalytic properties were studied using the catalytic hydration of α-pinene. The results showed that the composite catalyst of boric acid and tartaric acid had the highest catalytic activity. With an α-pinene, water, acetic acid, tartaric acid, and boric acid mass ratio of 10:10:25:0.5:0.4, the reaction temperature was 60 °C, the reaction time was 24 h, the conversion of α-pinene was 96.1%, and the selectivity of terpineol was 58.7%. The composite catalyst composed of boric acid and mandelic acid directly catalyzed the hydration of α-pinene in the absence of a solvent. Under the optimal conditions, the conversion of α-pinene reached 96.1%, and the selectivity of terpineol was 55.5%. When the composite catalyst catalyzed α-pinene to synthesize terpineol in one step, the terpineol was optically active, and terpineol synthesized using the two-step method with the dehydration of *p*-menthane-1,8-diol monohydrate was racemic. These composite catalysts may offer good application prospects in the synthesis of terpineol.

## 1. Introduction

Terpineol is an important spice. In addition to its use as a spice, terpineol also has antioxidant, anti-inflammatory, antiproliferative, antibacterial, and analgesic properties [1]. As early as 1947, Mosher studied α-pinene hydration products catalyzed by acids. In the industry, sulfuric acid with a concentration of 30% has been used to catalyze α-pinene to synthesize *p*-menthane-1,8-diol monohydrate (terpin hydrate) and then synthesize terpineol from terpin hydrate [2]. Terpineol synthesized via the dehydration of terpin hydrates includes a mixture of α-terpineol, β-terpineol, and γ-terpineol, with a ratio of about 7:2:2 [3]. The main problems with the two-step synthesis process of terpineol are its high energy consumption, serious equipment corrosion, and large wastewater treatment capacity requirements.

To solve the environmental pollution caused by the two-step synthesis of terpineol, one-step methods to synthesize terpineol from α-pinene have been developed. Román-Aguirre et al. used hydrochloric acid, acetic acid, oxalic acid, and monochloroacetic acid (MCA) as catalysts for the direct synthesis of terpineol from α-pinene [4]. Chloroacetic acid was found to be a good catalyst for the production of α-terpineol from pinene due to its strong acidity, high solubility, and high affinity for the aqueous and organic phases during the reaction. Wijayati et al. prepared a catalyst by impregnating trichloroacetic acid (TCA) on a Y-zeolite molecular sieve for the synthesis of terpineol from α-pinene [5]. Wijayati et al. also prepared a TCA/ZHY catalyst via impregnation to achieve terpineol synthesis [6], and Ávila et al. prepared catalysts for α-pinene hydration by impregnating TCA on SiO_2_, TiO_2_, and ZrO_2_·nH_2_O [7]. Sekerová et al. treated montmorillonite K10 with several acids (H_2_SO_4_, HCl, HNO_3_, and MCA) to catalyze the hydration of α-pinene [8], and Comelli et al. prepared a catalyst for α-pinene hydration by impregnating natural clay with MCA [9]. Because monochloroacetic acid is soluble in both α-pinene and water, its catalytic effect was found to be better than that of sulfuric acid [5,10]. When chloroacetic acid is not used, the yield of terpineol is not high. When using isopropanol as a solvent and macroporous cation exchange resin as a catalyst, the yield of terpineol was found to be about 36.5% [11]. With acetone as a solvent, the hydration of α-pinene was catalyzed by a polydimethylsiloxane (PDMS) membrane loaded with a USY molecular sieve. The reaction time reached 150 h, and the yield of terpineol was about 51% [12].

Because chloroacetic acid is highly corrosive and toxic, we studied the catalytic hydration of α-pinene with α-hydroxycarboxylic acid (HCA) [13]. We found that the reaction rate of α-pinene was very slow when HCA was used as a catalyst alone. A composite catalyst composed of HCA and phosphoric acid improved the conversion of α-pinene, but the yield of terpineol still needed improvement. Boric acid could form complexes with compounds containing hydroxyls, forming 1:1 and 1:2 complexes with glycolic and tartaric compounds [14]. When tartaric acid and boric acid formed complexes, the conductivity and optical rotation increased [15,16]. 

To improve the yield of terpineol, HCA and boric acid were used as composite catalysts to study the effects of their composition changes on catalytic performance, and catalysts with high catalytic activity were selected. Based on a screening of the catalysts, the synthesis of terpineol without an organic solvent and using the two-step synthesis of terpineol were explored. The mechanism of α-pinene hydration catalyzed by the composite catalysts was also studied. Because HCA and boric acid were easily soluble in water, the catalyst and water were in the lower layer and the product was in the upper layer after the reaction. Then, we used the byproduct of the pinene hydration reaction as an additive in the reaction, and the results showed that the selectivity of terpineol was improved [17]. Thus, the acid water containing the catalyst in the lower layer could be directly used for the next reaction.

## 2. Results and Discussion

Although terpineol is widely available in plant essential oils, commercial terpineol is mainly obtained through synthesis [1], making it meaningful to explore the use of HCA–boronic acid complexes as catalysts for the synthesis of terpineol from α-pinene.

### 2.1. Effect of the HCA–Boronic Acid Mixture Composition on Catalytic Activity

**Figure 1:**
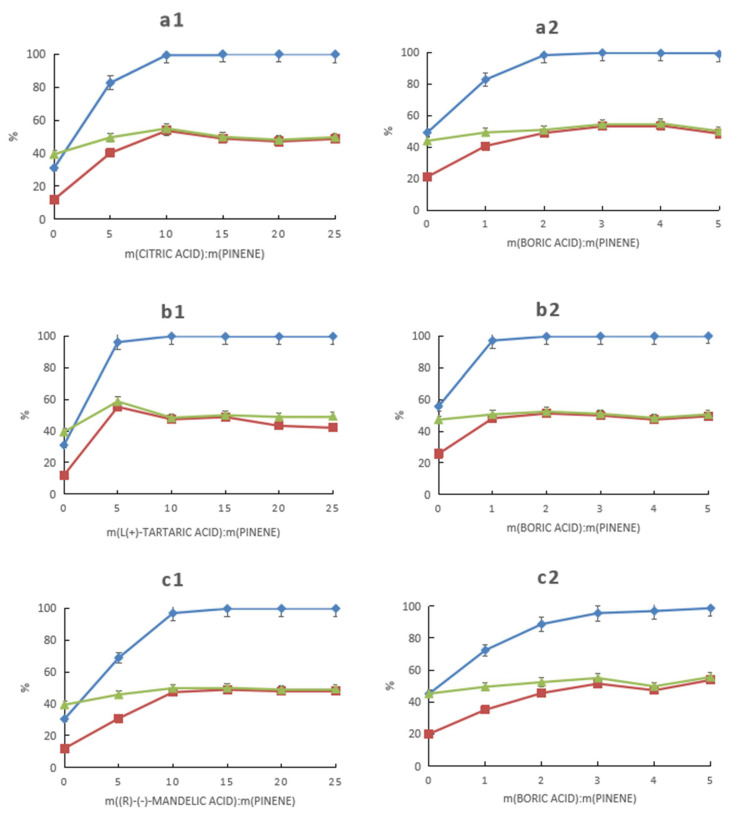

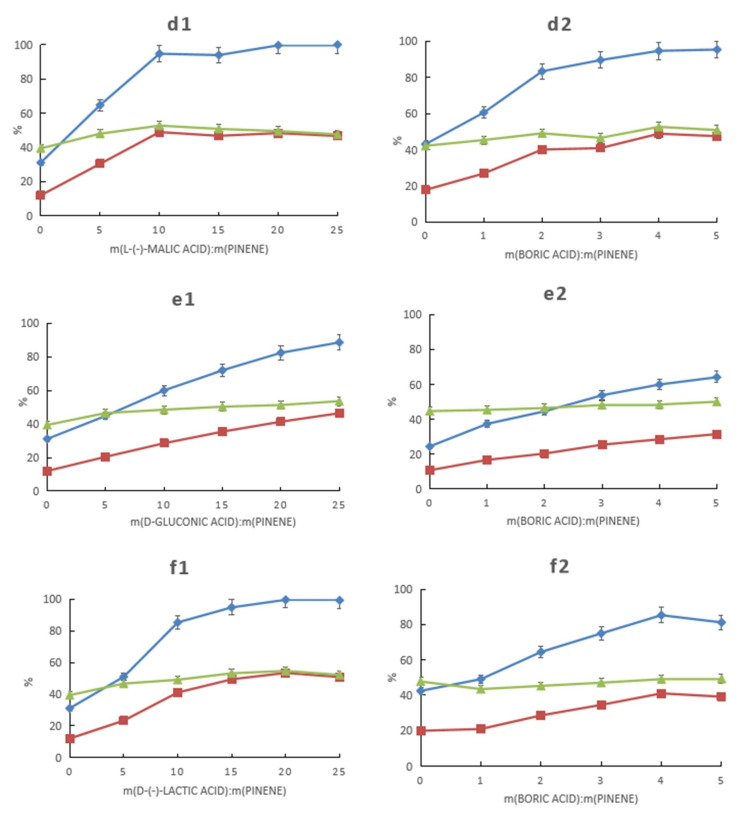

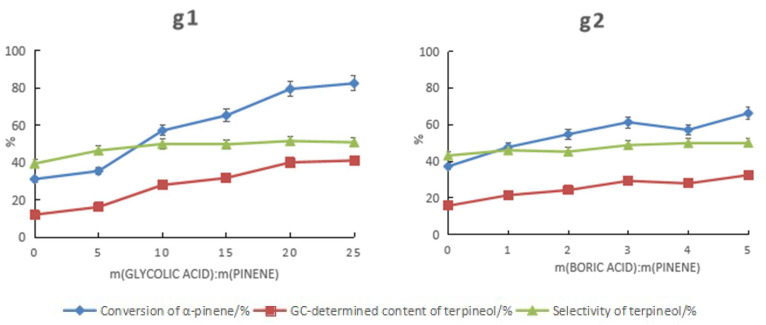
Effect of the HCA–boronic acid mixture composition on the conversion of α-pinene and the GC-determined content and selectivity of terpineol. (**a**–**g**) refer to citric acid, l-(+)-tartaric acid, dl-mandelic acid, l-(−)-malic acid, d-gluconic acid, d-(−)-lactic acid, and glycolic acid, respectively. The graphs on the left (**a1**–**g1**) show the effects of HCAs on catalytic performance under the following reaction conditions: m(α-pinene)/m(water)/m(acetic acid)/m(boric acid) = 10:10:25:0.4, reaction temperature of 60 °C, and a reaction time of 24 h. The graphs on the right (**a2**–**g2**) show the effects of boric acid on catalytic performance under the following reaction conditions: m(α-pinene)/m(water)/m(acetic acid)/m(HCAs) = 10:10:25:1, reaction temperature of 60 °C, and a reaction time of 24 h.

As described by Meng et al. [13], HCAs can catalyze the synthesis of α-pinene to terpineol but have low catalytic activity. Thus, the use of H_2_SO_4_ or H_3_PO_4_ is needed to improve the catalytic activity. Boric acid is a weak acid, and the conversion of α-pinene is only 30.5% when using boric acid alone as the catalyst, with a terpineol content of only 12%, as determined by GC (Figure 1(a1–g1)). However, boric acid can form complexes with HCAs, which have a higher ability to donate protons than HCAs, thus improving the catalytic activity (Figure 1(a2–g2)).

The catalytic activity of the catalysts for the hydration of α-pinene was positively correlated with the pH values of the aqueous solutions of the complexes. The optimal dosage of each group of catalysts, under the given experimental conditions, is shown in Table 1 as a mass ratio relative to α-pinene. Citric, tartaric, mandelic, and malic acids were used at mass ratios of no more than 10%, and their corresponding complexes with boric acid showed relatively high catalytic activity, while gluconic, lactic, and glycolic acids had to be used at mass ratios of at least 20% because their corresponding complexes with boric acid had relatively low catalytic activity. All catalysts showed selectivity above 50%, with the highest being 58.7% for the catalyst corresponding to tartaric acid. The lowest dosage of the seven HCAs was 5% for tartaric acid, whose boric acid complex had the highest catalytic activity at a molar ratio of about 2:1 of boric acid to tartaric acid.

The synthesis of α-pinene to terpineol is accompanied by the formation of isomers such as camphene, limonene, and terpinolene as side reactions. Boronic acid complexes with HCAs as catalysts can balance the conversion of α-pinene and the selectivity of terpineol. As a very distinctive type of catalyst, the boronic acid and HCAs were synergistic with each other. It is evident in Figure 1e and g that the conversion of α-pinene and the selectivity of terpineol gradually increased when either the dosage of the HCAs or that of boric acid increased.

### 2.2. Effects of Acetic Acid, Water, and Reaction Temperature on α-Pinene Hydration

**Figure 2:**
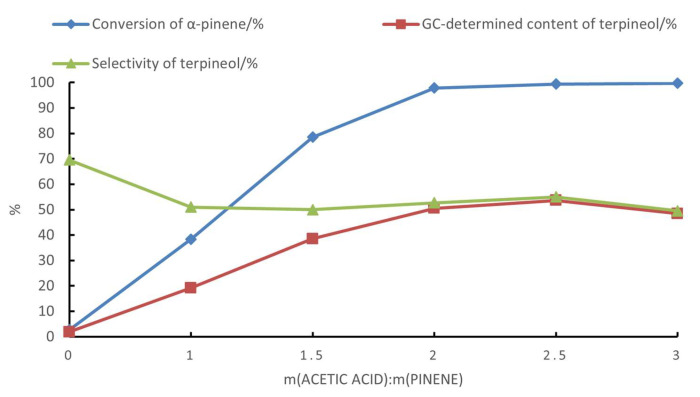
The effect of acetic acid on the hydration of α-pinene. The reaction conditions were m(α-pinene)/m(water)/m(citric acid)/m(boric acid) = 10:10:1:0.4, a reaction temperature of 60 °C, and a reaction time of 24 h.

**Figure 3:**
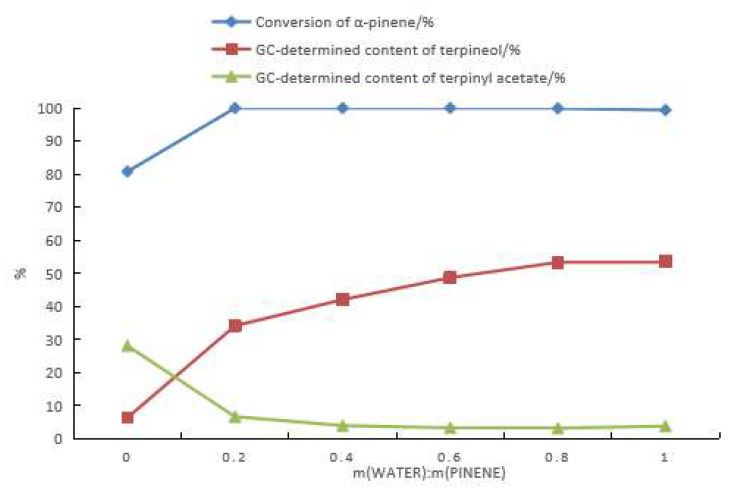
The effect of water on the hydration of α-pinene with the following reaction conditions: m(α-pinene)/m(acetic acid)/m(citric acid)/m(boric acid) = 10:25:1:0.4, a reaction temperature of 60 °C, and a reaction time of 24 h.

**Figure 4:**
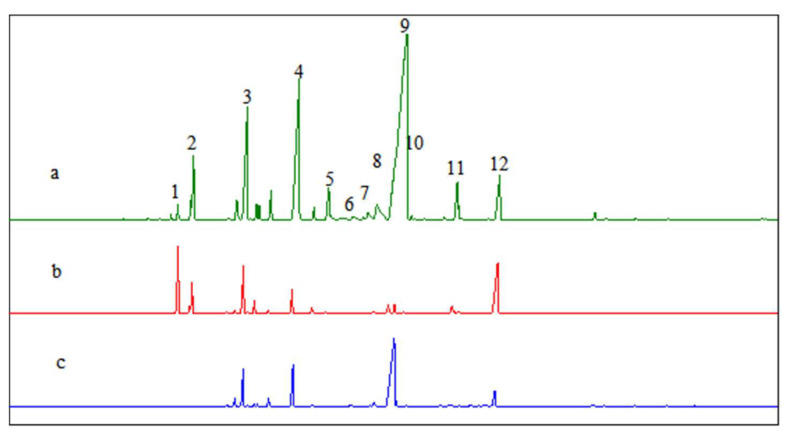
GC chromatograms of the reaction products: (a) with water in the starting material; (b) without water in the starting material; and (c) with terpineol as the starting material. Note: 1. α-pinene; 2. camphene; 3. limonene; 4. iso-terpinene; 5. fenchyl alcohol; 6. β-terpineol; 7. (iso)borneol; 8. 4-terpineol; 9. α-terpineol; 10. γ-terpineol; 11. bornyl acetate; 12. terpinyl acetate.

The effects of the acetic acid and water dosages on the hydration of α-pinene were investigated by employing boric acid–citric acid complexes as catalysts (Figure 2, Figure 3 and Figure 4). As shown in Figure 2, the hydration of α-pinene in the absence of acetic acid was difficult; after 24 h, the conversion of α-pinene was less than 3% at 60 °C. With an increase in the acetic acid dosage, the conversion of α-pinene and the content of terpineol gradually increased, as determined by GC. However, the selectivity of terpineol decreased when the mass ratio of acetic acid to α-pinene exceeded 2.5.

As shown in Figure 3, the content of terpinyl acetate among the products decreased and the content of terpineol increased with an increasing water dosage in the reaction mixture. Figure 4 demonstrates the GC chromatograms of the products obtained under the same reaction conditions in the three scenarios: the presence of water in the starting material (scenario **a**), the absence of water in the starting material (scenario **b**), and the use of terpineol as the starting material (scenario **c**) (Figure 4). Specifically, Figure 4a shows that when the starting material contained water, the products were dominated by pinenol. Figure 4b shows that when the starting material was anhydrous, the products were dominated by terpinyl acetate. Figure 4c shows that when the starting material was pinenol, the compositions of the products were similar to those in scenario **a,** except for the absence of camphene, fenchol, borneol, and bornyl acetate (formed by the isomerization of α-pinene). The products in scenarios **b** and **c** contained limonene, terpinene, terpineol, and terpinyl acetate, with the same relative content of terpineol as in scenario **a**.

**Figure 5:**
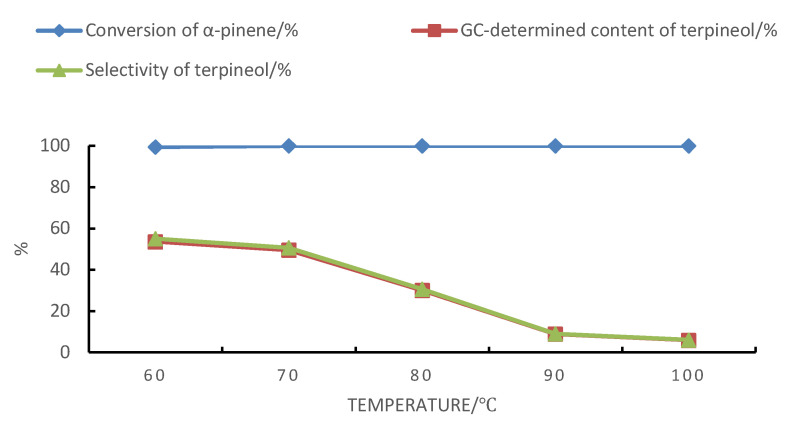
Effect of temperature on the hydration of α-pinene with the following reaction conditions: m(α-pinene)/m(water)/m(acetic acid)/m(citric acid)/m(boric acid) = 10:10:25:1:0.4, 24 h.

The reaction temperature had a great influence on the α-pinene hydration reaction, as shown in Figure 5. As presented in Figure 5, when using boric acid–citric acid complexes to catalyze the hydration of α-pinene, the selectivity of terpineol decreased significantly at temperatures above 70 °C. Meanwhile, the conversion of α-pinene was 99.4% at 60 °C and 100% at 70 °C, and the high consistency between the content and selectivity trends of the product terpineol indicated that the decrease in the selectivity with increasing temperatures could be attributed to the dehydration of terpineol.

### 2.3. Analysis of the α-Pinene Hydration Products

**Figure 6:**
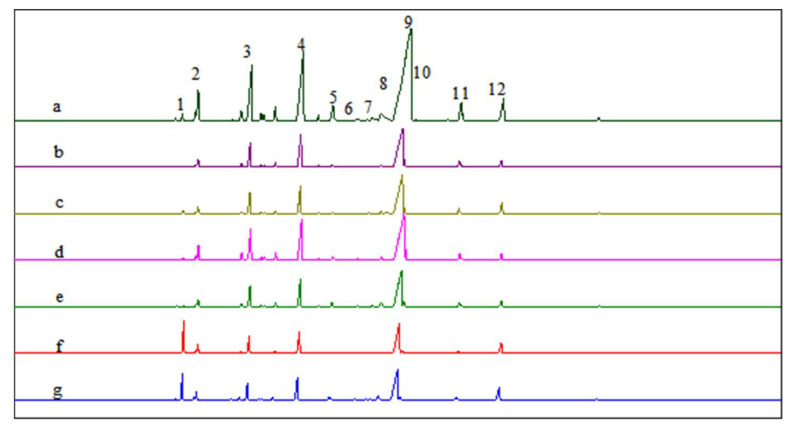
GC chromatograms of the α-pinene hydration products when boric acid–HCA complexes were used as the catalyst: (a) citric acid; (b) l(+)-tartaric acid; (c) dl-mandelic acid; (d) l-(−)-malic acid; (e) d-(−)-lactic acid; (f) d-gluconic acid; (g) glycolic acid. Note: 1. α-pinene; 2. camphene; 3. limonene; 4. iso-terpinene; 5. fenchyl alcohol; 6. β-terpineol; 7. (iso)borneol; 8. 4-terpineol; 9. α-terpineol; 10. γ-terpineol; 11. bornyl acetate; 12. terpinyl acetate.

To determine whether there were significant differences in product distribution as a function of the catalyst, the GC chromatograms of the products listed in Table 1 were arranged vertically in Figure 6. No significant differences were observed among the catalysts in terms of product composition and content. The α-pinene hydration under the catalysis of boronic acid complexes with HCAs followed the same reaction mechanism. As indicated in a previous study [13], l-terpineol could be obtained by using l-α-pinene as the starting material, and d-terpineol could be obtained by using d-α-pinene as the starting material. In addition to isomeric byproducts, terpinyl acetate was also a significant byproduct. As shown in Figure 6b, the total content of terpineol and terpinyl acetate was 63.6% (determined by GC) when the boric acid–tartaric acid complex was used as the catalyst.

The terpineol synthesized using the one-step method was subjected to vacuum fractionation, and the GC content of the terpineol was 98%. The ^1^H NMR and ^13^C NMR spectra of the terpineol are included in the Supplementary Materials (Figures S1 and S2). The NMR results were as follows: ^1^H NMR (300 MHz, CDCl_3_) δ 5.38 (d, *J* = 3.0 Hz, 1H), 2.09–1.97 (m, 4H), 1.65 (s, 3H), 1.57–1.45 (m, 3H), and 1.18 (d, *J* = 3.0 Hz, 6H), and ^13^C NMR (75 MHz, CDCl_3_) δ 134.05, 120.54, 44.99, 31.01, 27.44, 26.89, 26.25, 23.97, 23.37, and 23.12. The infrared spectrum of terpineol is shown in the Supplementary Materials (Figure S3). Absorption was observed at 3395.57 cm^−1^, 1366.88 cm^−1^, and 1132.58 cm^−1^, which corresponded to the OH stretching vibration, the in-plane deformation vibration of the tertiary alcohol δ(OH), and the stretching vibration of the tertiary alcohol ν(C–O), respectively (Figure S3). At 3395.57 cm^−1^, the peak shape was broad due to the hydrogen bond association between the terpineol molecules.

### 2.4. Direct Catalytic Hydration of α-Pinene without a Solvent

To reduce mass transfer resistance during the α-pinene hydration reaction, acetone, acetic acid, and other solvents must be added as auxiliaries, increasing the cost of solvent recovery. Interestingly, the catalyst composed of boric acid and mandelic acid was able to directly catalyze the reaction of α-pinene with water to obtain terpineol without any solvent as the auxiliary. Under the reaction conditions of m(α-pinene)/m(water)/m(mandelic acid)/m(boric acid) = 1:3.5:0.3:0.03, 60 °C, and 20 h, the conversion of α-pinene reached 96.1%, and the selectivity of terpineol was 55.5%. The benzene ring contained in the mandelic acid molecule made it easier to dissolve in α-pinene, and the hydroxyl and carboxyl groups contained in the mandelic acid molecule readily combined with water. Under the reaction conditions of m(α-pinene)/m(water)/m(HCA)/m(boric acid) = 1:1.5:0.3:0.03, 60 °C, and 20 h, the conversion rates of α-pinene were 96.1%, 83.5%, 43.8%, 22.7%, 7.6%, 6.1%, and 4.6% when the utilized HCAs were mandelic acid, lactic acid, malic acid, citric acid, tartaric acid, gluconic acid, and glycolic acid, respectively, as shown in Table 2. Therefore, the composite catalyst composed of mandelic acid and boric acid could directly catalyze the hydration reaction of pinene, while other HCAs had poor catalytic performances. At the same time, we used phosphoric acid as the catalyst to carry out comparative experiments. The catalytic performance of phosphoric acid was poor, and the pinene conversion rate was only 5.7% (Table 2).

In the one-step synthesis of terpineol from α-pinene catalyzed by HCA–boric acid, terpineol had optical activity, l-pineol was produced when l-pinene was used, and d-terpineol was produced when d-pinene was used. The enantiomeric excess (ee) value of the terpineol was 71%, similar to the value reported in [13], where the ee value of terpineol was about 70%. The results of the α-pinene hydration reaction catalyzed by HCA and boric acid in the solvent-free condition are shown in Table 2, indicating that the mandelic acid–boric acid composite catalyst had the best catalytic effect, followed by the lactic acid–boric acid composite catalyst, while the glycolic acid–boric acid composite catalyst had the worst catalytic effect. Compared with glycolic acid, lactic acid had one more methyl group, but under the same reaction conditions, the conversion of α-pinene to lactic acid was 20-fold higher than that of glycolic acid. At present, no other catalysts have been found with similar catalytic effects.

### 2.5. Terpin Hydrate Synthesis

When 30% sulfuric acid was used to catalyze the reaction of α-pinene and water to synthesize terpin monohydrate, large refrigeration equipment was required for industrial production due to the exothermic reaction, resulting in large power consumption. The composite catalyst composed of HCA and boric acid could catalyze the reaction of α-pinene and water to synthesize terpin monohydrate, and the reaction process did not undergo intense heat release. Synthesis with the tartaric acid–boric acid catalyst was performed under the following conditions: a mass ratio of α-pinene, water, tartaric acid, and boric acid of 7:13:7:4, a reaction temperature maintained at 20–25 °C, and a reaction time of 50 h. After the reaction, the product was crystallized at room temperature. Then, the terpin monohydrate was separated. The yield of terpin monohydrate was 110% by weight and 88% by mol.

Compared to sulfuric acid, the crystal particles of terpin monohydrate synthesized from α-pinene catalyzed by the tartaric acid–boric acid composite catalyst were finer, and they were white. When terpin monohydrate was dehydrated to synthesize terpineol, the concentration of sulfuric acid in the aqueous solution had to be maintained at 0.1% because the yield would be reduced when the concentration of acid was higher. When sulfuric acid was used as the catalyst, the hydrated crystal of terpin monohydrate contained sulfuric acid, which, if not neutralized, would affect the next dehydration reaction and reduce the yield of terpineol. When tartaric acid–boric acid was used as the catalyst, the hydrate crystal would not need to be neutralized. The terpin monohydrate dehydration reaction did not require an additional catalyst, and its residual catalyst could play a catalytic role, with the yield of terpineol reaching 85%. The terpineol obtained via the two-step process of α-pinene catalyzed by a tartaric acid–boric acid catalyst was not optically active (see the Supplementary Materials for the GC spectra of terpin monohydrate and terpineol prepared after terpin monohydrate dehydration (Figures S4 and S5)).

### 2.6. Study on the Hydration Reaction Mechanism

**Figure 7:**
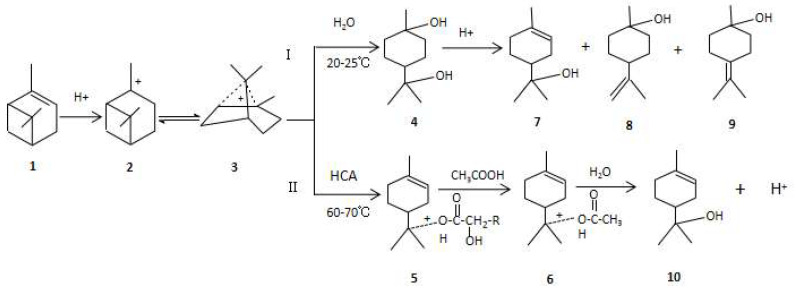
Hydration of α-pinene catalyzed by the HCA–boric acid complex catalyst. **1**: α-Pinene; **2**: carbocation; **3**: carbocation; **4**: *p*-menthane-1,8-diol monohydrate; **5**: oxonium ion terpinyl hydroxycarboxylate; **6**: oxonium ion terpinyl acetate; **7**: dl-α-terpineol; **8**: β-terpineol; **9**: γ-terpineol; **10**: α-terpineol.

The reaction process of the α-pinene synthesis of terpineol catalyzed by HCA–boric acid was as follows: The double bond of α-pinene was attacked by hydrogen ions to form carbocation **2**, and carbocation **2** was rearranged to obtain carbocation **3**. Then, according to the different reaction conditions, terpineol was obtained through paths I and II, as shown in Figure 7.

Reaction path I required excessive water, a high [H^+^] concentration, a low reaction temperature, and a long reaction time, and the reaction product was terpin hydrate **4**. Then, terpin hydrate was dehydrated under acid catalysis to obtain dl-α-terpineol, β-terpineol, and γ-terpineol.

Reaction path II required a higher reaction temperature, a lower two-phase mass transfer resistance, and a shorter reaction time. The terpineol synthesized using the one-step method was mainly α-terpineol, and the product had optical activity.

First, carbocation **3** and HCA formed the intermediate of oxonium ion terpinyl hydroxycarboxylate **5**, and then, if acetic acid was added to the raw material, it was converted to the intermediate of oxonium ion terpinyl acetate and then hydrolyzed to terpineol. By using lactic acid and boric acid to catalyze the reaction of pinene with water, terpinyl lactate could be detected in the synthesized product (see Supplementary Materials (Figure S6)). If acetic acid was not added to the reaction system, the reaction rate was slow due to the high mass transfer resistance of the oil–water phase. However, when mandelic–boric acid was used, terpineol could be successfully synthesized by reducing the amount of water without adding other organic solvents to improve the two-phase mass transfer. Terpineol was synthesized from α-pinene in one step with the HCA–boric acid catalyst. The content of terpineol in the product could reach 55.3%, which was 45% higher than the current yield of chiral terpineol [18]. When HCA–boric acid was used to catalyze the hydration of pinene, reaction paths I and II existed simultaneously. Due to the formation of a small amount of terpin monohydrate and further dehydration to obtain racemic terpineol, the ee value of the one-step synthesis of terpineol was lower than that of α-pinene.

## 3. Methods and Materials

### 3.1. Materials and Apparatus

The following starting materials and reagents were used in this study: (−)-α-pinene (98%), (+)-α-pinene (98%, ee 92%), terpinyl acetate (97%), α-terpineol (98%), glycolic acid (98%), citric acid (99.5%), l-(+)-tartaric acid (99.5%), d-(−)-tartaric acid (99.5%), d-(−)-lactic acid (90%), (*R*)-(−)-mandelic acid (99%), dl-mandelic acid (99%), l-(−)-malic acid, and d-gluconic acid (49–53 wt.% in H_2_O). These compounds were purchased from Macklin and Aladdin (Shanghai, China). Acetic acid (99.5%), sodium hydroxide (98%), and sodium carbonate anhydrous (98%) were purchased from Chengdu Kelong Chemical (Chengdu, Sichuan Province, China). Distilled water was made in the laboratory.

The reaction apparatus was a PPV-3000 organic synthesis unit (EYELA, Tokyo Rikakikai, Tokyo, Japan). The following analytical instruments were used in this work: a near-infrared quality analyzer (SY-3650-II, FOSS NIRSystems Inc., Hilleroed, Denmark); an AVANCE III 300M nuclear magnetic resonance spectrometer (Bruker, Fällanden, Switzerland); a 7890A gas chromatograph (Agilent, Santa Clara, CA, USA) equipped with quartz capillary chromatography columns (60 m × 0.25 mm × 0.25 μm) with AT-35 as the immobile phase; and a TQ456 GC-MS instrument (Bruker, Billerica, MA, USA) equipped with BR-5 elastic quartz capillary columns (30 m × 0.25 mm × 0.25 μm) as chromatography columns. A Thermo Nicolet iS50 Fourier transform infrared spectrometer (Thermo Fisher Scientific, Waltham, MA, USA) was used.

### 3.2. Synthetic Procedures

Terpineol synthesis: In a reaction flask, 10 g of α-pinene, 25 g of acetic acid, 10 g of water, 0–0.5 g of boric acid, and 0–2.5 g of HCA were added and stirred using magnetic stirring (500 rpm). The reaction temperature was maintained at 60 °C, and the reaction time was 24 h. After the reaction, the product was poured into a separatory funnel and allowed to settle into two distinct layers, with the upper layer consisting of the product layer and the lower layer consisting of an aqueous acetic acid solution containing the catalyst. The upper layer was neutralized with 0.5 M NaOH, washed with water, dried with anhydrous sodium sulfate, and then sampled for analysis.

Solvent-free synthesis of terpineol: In a reaction flask, 10 g of α-pinene, 3.5 g of water, 3 g of mandelic acid, and 0.3 g of boric acid were added and stirred using magnetic stirring (500 rpm). The reaction temperature was maintained at 60 °C, and the reaction time was 50 h. After the reaction, the product was poured into a separatory funnel, and water was added to settle into two distinct layers. The upper layer was neutralized with 0.5 M NaOH, washed with water, dried with anhydrous sodium sulfate, and then sampled for analysis.

Terpin hydrate synthesis: In a reaction flask, 50 g of α-pinene, 70 g of water, 35 g of tartaric acid, and 28 g of boric acid were added and stirred using electric stirring (500 rpm). The reaction temperature was maintained at 20–25 °C, and the reaction time was 50 h. After the reaction, the product was poured into a beaker and allowed to crystallize at room temperature. The terpin hydrate was collected via filtration and neutralized with 2 M NaOH. After neutralization, the coarse crystal was washed, filtered, and dried to obtain terpin hydrate.

### 3.3. Analytical Methods

The proportions of the starting materials and products were calculated using the gas chromatography (GC) area normalization method. The conversion of α-pinene and terpineol selectivity were estimated according to the following formulas:

(1) $$\mathrm{Conversion}\;\mathrm{of}\;\mathrm{pinene}=\frac{\mathrm{GC}\text{-}\mathrm{determined}\;\mathrm{content}\;\mathrm{of}\;\mathrm{pinene}\;\mathrm{before}\;\mathrm{reaction}-\mathrm{GC}\text{-}\mathrm{determined}\;\mathrm{content}\;\mathrm{of}\;\mathrm{pinene}\;\mathrm{after}\;\mathrm{reaction}}{\mathrm{GC}\text{-}\mathrm{determined}\;\mathrm{content}\;\mathrm{of}\;\mathrm{pinene}\;\mathrm{before}\;\mathrm{reaction}},$$

(2) $$\mathrm{Terpineol}\;\mathrm{selectivity}=\frac{\mathrm{GC}\text{-}\mathrm{determined}\;\mathrm{content}\;\mathrm{of}\;\mathrm{terpineol}\;\mathrm{after}\;\mathrm{reaction}}{\mathrm{GC}\text{-}\mathrm{determined}\;\mathrm{content}\;\mathrm{of}\;\mathrm{pinene}\;\mathrm{before}\;\mathrm{reaction}-\mathrm{GC}\text{-}\mathrm{determined}\;\mathrm{content}\;\mathrm{of}\;\mathrm{pinene}\;\mathrm{after}\;\mathrm{reaction}}.$$

For the infrared spectrum (IR) data acquisition, the sample was placed on an infrared spectrometer slide with air as the background, and the spectrum was acquired in the wavenumber range of 400–4000 cm^−1^, where the number of sample scans was 24, the number of background scans was 24, the sample gain was 1.0, the mirror velocity was 0.6329, and the aperture was 95.00.

For the proton nuclear magnetic resonance (^1^H NMR) data acquisition, the sample was placed in a measuring sample tube, CDCl_3_ was added, and the sample was measured using a 300 MHz NMR instrument (frequency: 300 MHz), where the temperature was 296.9 K, the number of scans was 64, and the pulse width was 12.6 μs. The spectral width was 12,315.27, and the data point size was 32,768. The NMR spectra were processed using Mestrenova software and integrated after calibration, phase, and baseline calibration.

For the GC analysis, high-purity nitrogen was used as the carrier gas, and the temperature program was as follows: the initial temperature was 70 °C (2 min hold), with a first ramp of 5 °C/min to 150 °C (3 min hold), followed by a second ramp of 10 °C/min to 230 °C (10 min hold). The inlet temperature was set to 250 °C, and the total flow rate was set to 130.5 mL/min, with a split ratio of 50:1 and a septum purge rate of 3 mL/min. The analytes were detected using a flame ionization detector (FID) with a detection port temperature of 250 °C, a hydrogen flow rate of 40 mL/min, an air flow rate of 450 mL/min, and a nitrogen purge rate of 25 mL/min. The injection volume was 0.2 µL.

For the gas chromatography–mass spectrometer (GC-MS) analysis, high-purity helium was used as the carrier gas, and the temperature program was as follows: the initial temperature was 50 °C (3 min hold), with a first ramp of 20 °C/min to 120 °C, followed by a second ramp of 2 °C/min to 180 °C (2 min hold), and a third ramp of 50 °C/min to 250 °C (5 min hold). The inlet temperature was set to 230 °C, and the interface temperature was set to 250 °C.

For mass spectrometry, electron ionization (EI) was used as the ionization source, with an ionization voltage of 70 eV, where a full-scan mode was used with a scan range of 45–350 amu. In addition, a solvent delay time of 5 min was set, where the injection volume was set to 0.5 μL (the sample was dissolved in ethanol, with a mass fraction of 1%).

## 4. Conclusions

In the catalytic hydration of α-pinene, boric acid made a significant contribution to the catalytic activity of the HCAs and was superior to inorganic acids such as sulfuric acid and phosphoric acid for the conversion of α-pinene and selectivity to terpineol. The selectivity to terpineol in α-pinene hydration that was catalyzed by HCA–boronic acid complexes exceeded 50%, with the highest value being 58.7% for the catalyst system of tartaric acid. The composite catalyst composed of boric acid and mandelic acid could directly catalyze the hydration of α-pinene in the absence of a solvent. The conversion of α-pinene reached 96.1%, and the selectivity of terpineol was 55.5%. These types of complex catalysts are cost-effective, easy to obtain, and have low toxicity, giving them a promising application in the industrial production of terpineol.

Because mandelic acid contains a benzene ring, it is lipophilic. By increasing the amount of mandelic acid and reducing the amount of water, it could be catalyzed to synthesize terpineol in one step without the addition of organic solvents such as acetic acid. The conversion of α-pinene reached 96.1%, and the selectivity for terpineol was 55.5%.

The synthesis of terpin hydrate from α-pinene could be catalyzed by the tartaric acid–boric acid composite catalyst at 20–25 °C. Then, terpineol was prepared via the dehydration of terpin hydrate glycol, which contained the α, β, and γ isomers. The yield of terpin hydrate was 110% by weight and 88% by mol.

Due to the synergistic catalysis of HCA and boric acid, the conversion of α-pinene and the selectivity of terpineol improved. When using the HCA–boric acid composite catalyst to synthesize terpineol, the terpineol obtained using the one-step method had optical activity, and the terpineol obtained using the two-step method showed no optical activity.

## Figures and Tables

**Table 1:** Optimal dosages of boric acid and HCAs of each catalyst group *^a^*.

	HCA Dosage	Boric Acid Dosage	Conversion of α-Pinene (%)	GC-Determined Content of Terpineol (%)	Selectivity of Terpineol (%)
Citric acid	10%	4%	99.4	53.6	55.0
l-(+)-Tartaric acid	5%	4%	96.1	55.3	58.7
dl-Mandelic acid	10%	5%	98.7	53.8	55.6
l-(−)-Malic acid	10%	4%	94.7	48.9	52.7
d-Gluconic acid	25%	4%	88.5	46.4	53.6
d-(−)-Lactic acid	20%	4%	99.6	53.4	54.7
Glycolic acid	25%	4%	82.4	41.0	50.8

*^a^* The HCA dosage is expressed as the mass (m) ratio m(HCA)/m(α-pinene), and the dosage of boric acid is expressed as m(boric acid)/m(α-pinene). The reaction temperature was 60 °C, and the reaction time was 24 h.

**Table 2:** Results of α-pinene hydration catalyzed by HCA–boric acid without a solvent.

	HCA Dosage	Boric Acid Dosage	Conversion of α-Pinene (%)	GC-Determined Content of Terpineol (%)	Selectivity of Terpineol (%)
Citric acid	30%	3%	22.7	10.7	12
l-(+)-Tartaric acid	30%	3%	7.6	3.314	44.6
dl-Mandelic acid	30%	3%	96.1	53.3	55.5
l-(−)-Malic acid	30%	3%	43.8	20.8	48.5
d-Gluconic acid	30%	3%	6.1	0.4	53.6
d-(−)-Lactic acid	30%	3%	83.5	42.3	51.7
Glycolic acid	30%	3%	4.6	3.0	66.2
Phosphoric acid	30%	-	5.7	5.8	6.9

## Data Availability

The data presented in this study are available on request from the corresponding author.

## Supplementary Materials

The following supporting information can be downloaded at https://www.mdpi.com/article/10.3390/molecules28073202/s1, Figure S1: ^1^H NMR spectrum of terpineol; Figure S2: ^13^C NMR spectrum of terpineol; Figure S3: Infrared spectrum of the terpineol sample; Figure S4: The GC spectrogram of 1,8-terpenediol; Figure S5: Synthesis of terpineol by dehydration of 1,8-terpene diol; Figure S6: The GC spectrogram of the products from the solvent-free synthesis of terpineol using lactic acid–boric acid as a catalyst.
