# Supplementary material for: Study on the Hydration of α-Pinene Catalyzed by α-Hydroxycarboxylic Acid–Boric Acid Composite Catalysts
Source: Molecules. 2023 Apr 4;28(7):3202. doi: 10.3390/molecules28073202 (PMC10096544; doi:10.3390/molecules28073202)
Supplement: Supplementary file 1 [file molecules-28-03202-s001.zip › molecules-2254748-supplementary.pdf]

Supporting Materials

Study on the Hydration of α-Pinene Catalyzed by α-Hydroxycarboxylic-boric Acid Composite Catalysts

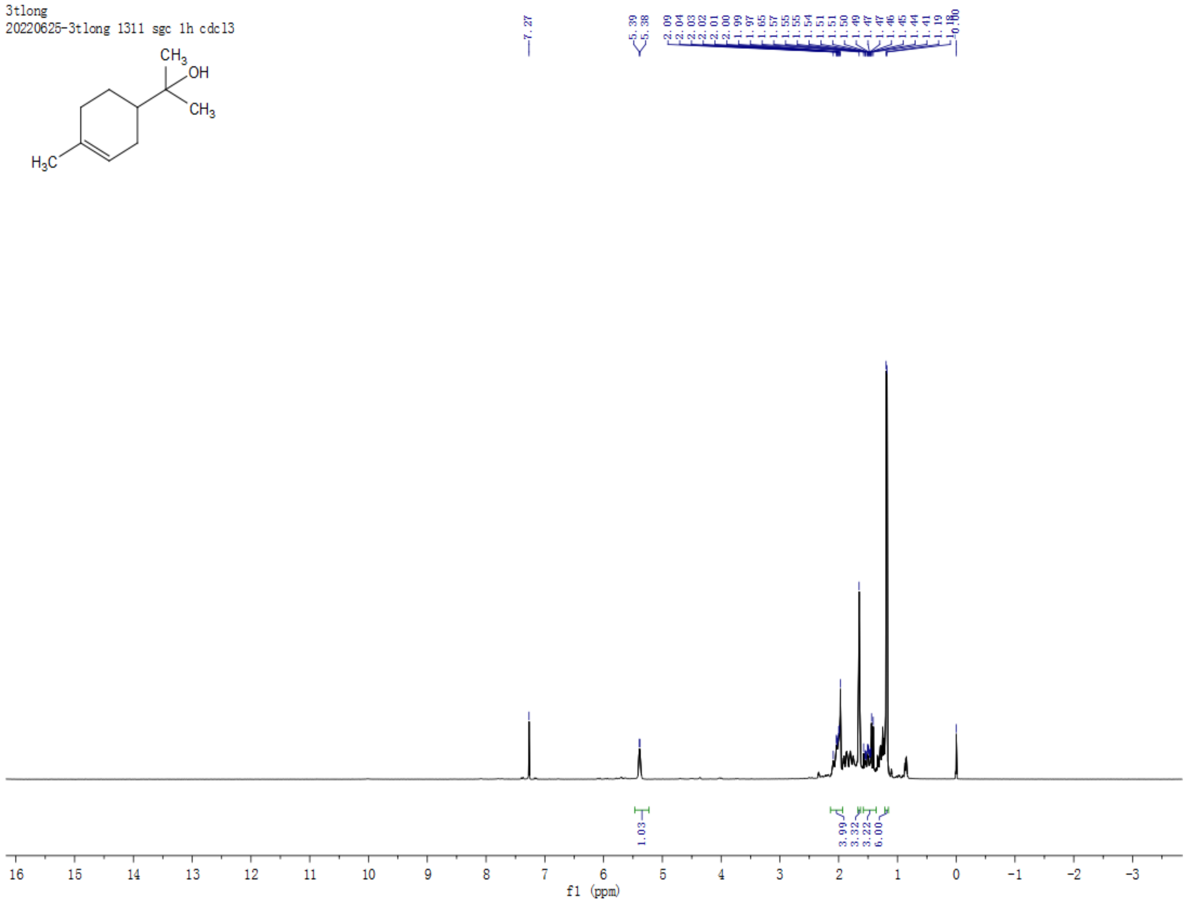

Figure S1. ^{1}H NMR spectrum of terpineol.

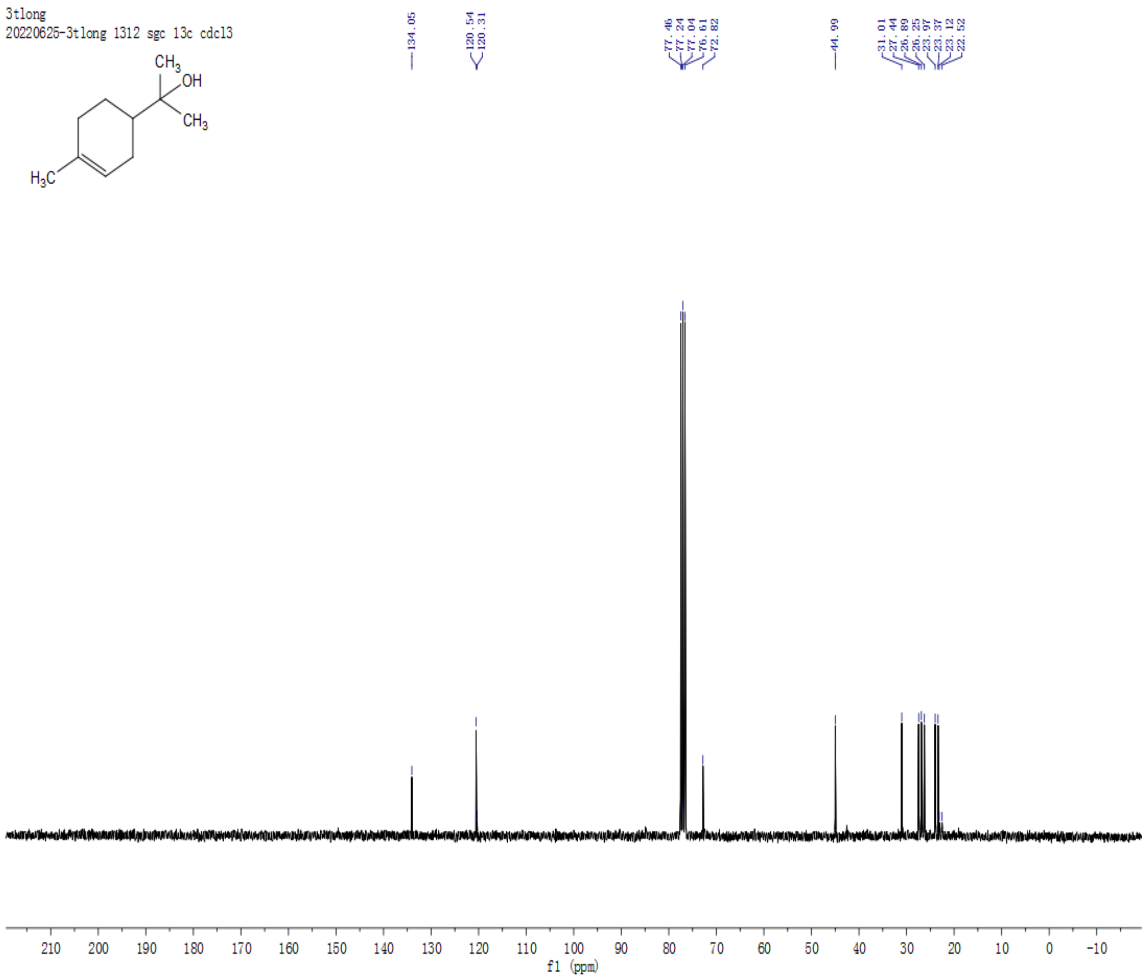

Figure S2. ^{13}C NMR spectrum of terpineol.

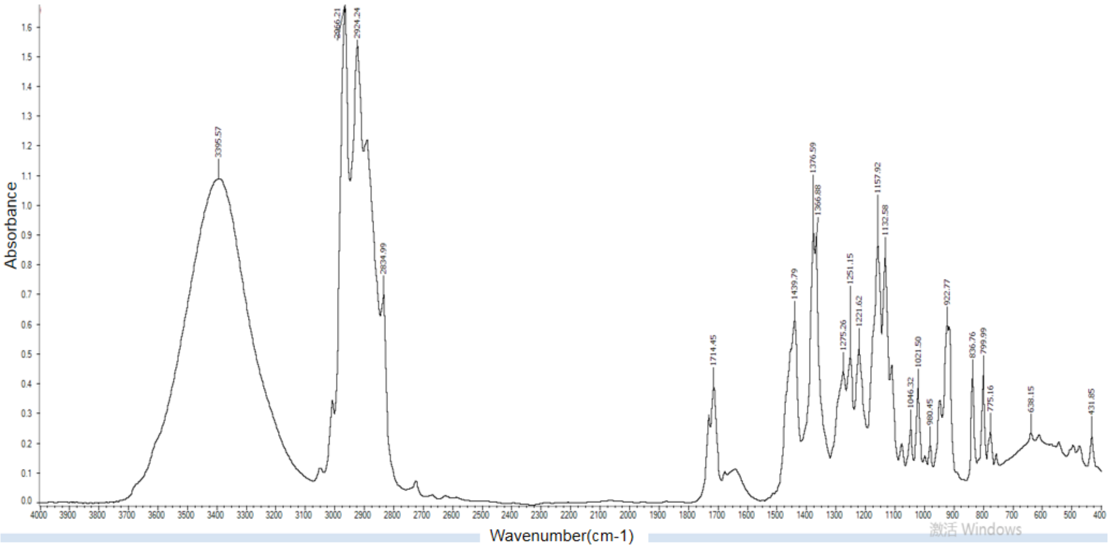

Figure S3. Infrared spectrum of terpineol sample.

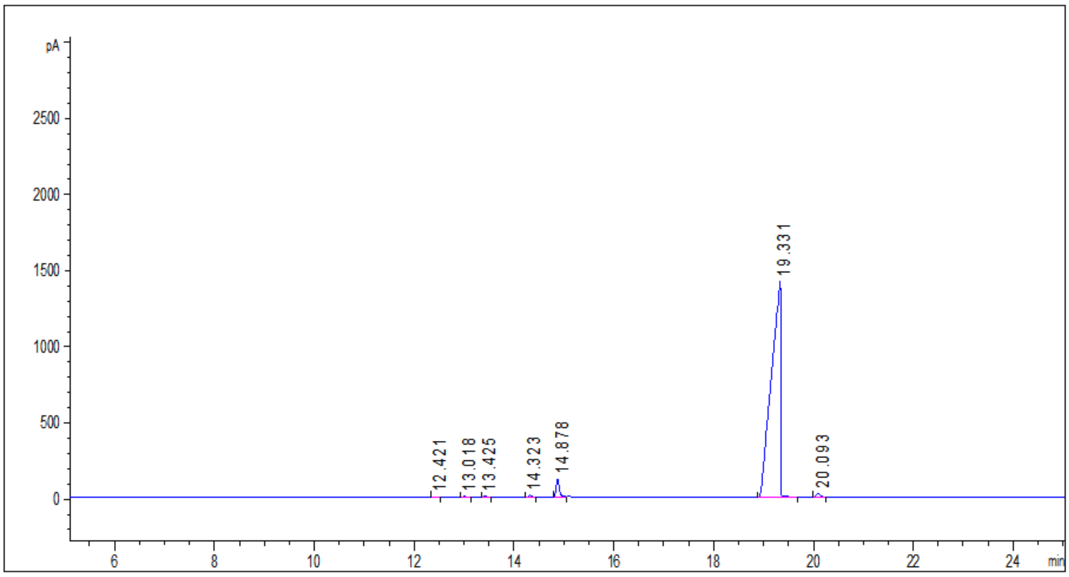

Figure S4. The GC Spectrogram of 1,8-Terpenediol.

Note: 14.876 min is terpineol; 19.331 min for 1,8-terpene diol.

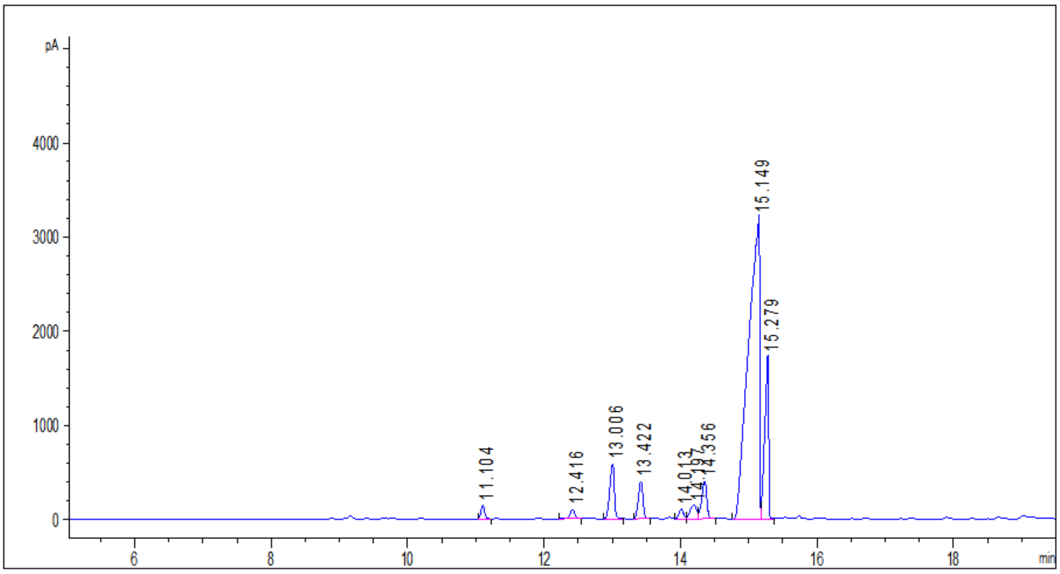

Figure S5. Synthesis of terpineol by dehydration of 1,8-terpene diol.

Note: 13.006 min is β-Terpineol; 14.356 min is 4-terpineol; 15.149 min α-Terpineol; 15.279 min is γ- Terpineol.

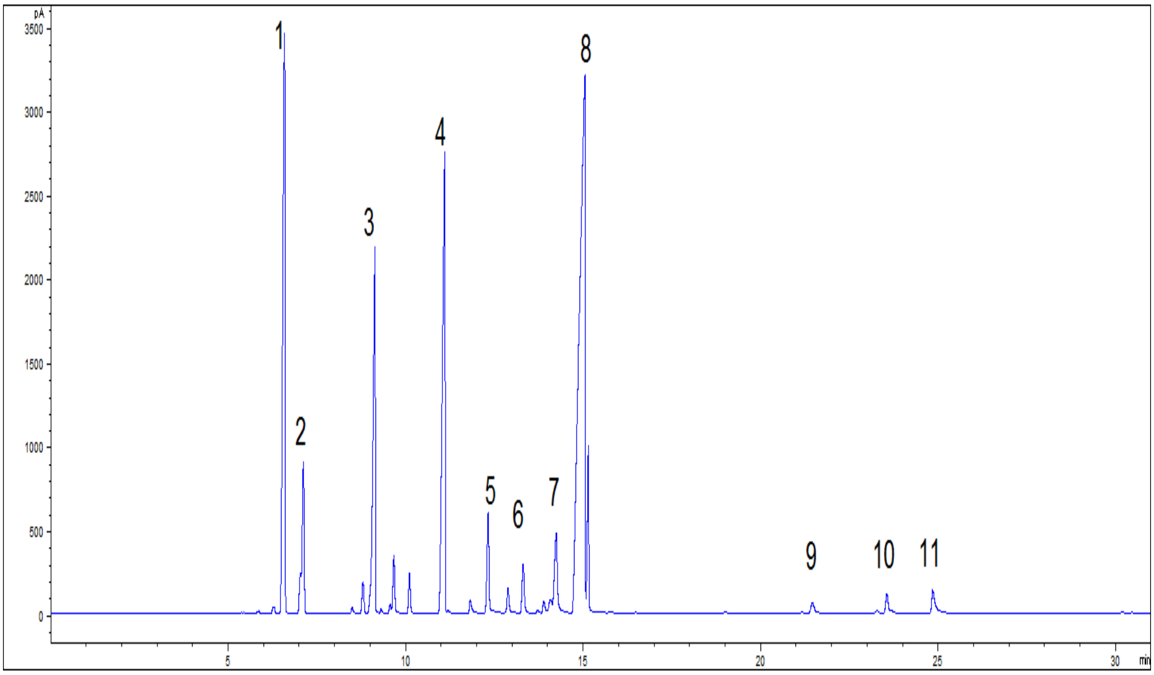

Figure S6. The GC Spectrogram of the Products from the Solvent Free Synthesis of Terpineol Using Lactic Acid Boric Acid as Catalyst.

Note: 1. Pinene; 2. Camphene; 3. Limonene; 4. Terpinene; 5. Fenpropanol; 6. β-Terpineol; 7.4 Terpineol; 8. α-Terpineol; 9. fenvalerate lactate; 10. Bornyl lactate; 11. Terpinyl lactate.
